# Supplementary material for: Human transcription factor and protein kinase gene fusions in human cancer
Source: Sci Rep. 2020 Aug 25;10:14169. doi: 10.1038/s41598-020-71040-8 (PMC7447636; doi:10.1038/s41598-020-71040-8)
Supplement: Supplementary file 1 — Supplementary Legends. [file 41598_2020_71040_MOESM1_ESM.docx]

**Supplementary figure S1**

**A)** Stage and project distribution of all oncofusions found in the TCGA dataset. **B)** Distribution of protein producing oncofusions across TCGA cancer projects. Distribution seen is nearly identical to that seen with all fusions, and similar to that of PK/TF fusions (figure 2A).

**Supplementary figure S2**

Chromosomal distributions of fusion breakpoints as percentage of chromosome length.

**A)**  PK/TF fusions feature one very prominent hotspot around the 50 % mark of chromosome 17, and several less intense ones. The 50 % spot on chromosome 17 is driven mainly by oncofusions with either ERBB2 or RARA as one fusion gene. **B)** Protein producing fusion breakpoints feature prominent hotspots in common with PK/TF fusions, and in addition several unique spots, where not many PK/TF fusions are present.

**Supplementary figure S3**

Distribution of intact, full-length domains in fusion proteins per TCGA project. Counts are minmax normalized to 1 per project to account for variable number of fusions in each project.
