# Supplementary material for: Human transcription factor and protein kinase gene fusions in human cancer
Source: Sci Rep. 2020 Aug 25;10:14169. doi: 10.1038/s41598-020-71040-8 (PMC7447636; doi:10.1038/s41598-020-71040-8)

Supplementary figures for Human Transcription Factor and Protein Kinase Gene Fusions in Human Cancer

Kari Salokas[1], Rigbe G. Weldatsadik[1] and Varjosalo Markku[1*]

[1]Systems Pathology/Biology Research Group, Institute of Biotechnology, HiLIFE, University of Helsinki, Helsinki, Finland.

* Correspondence: markku.varjosalo@helsinki.fi

Supplementary figure 1

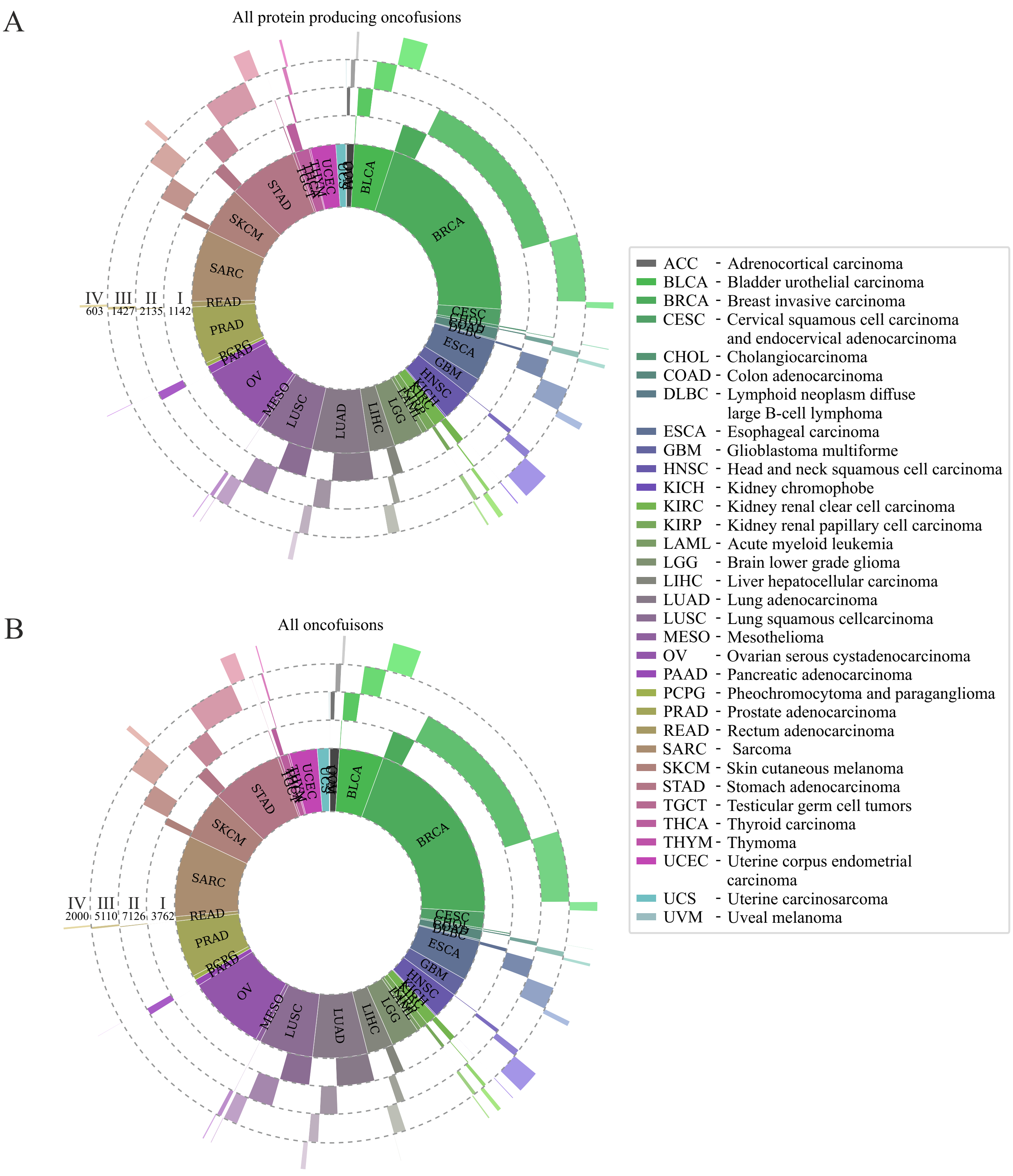

Supplementary figure 2

A

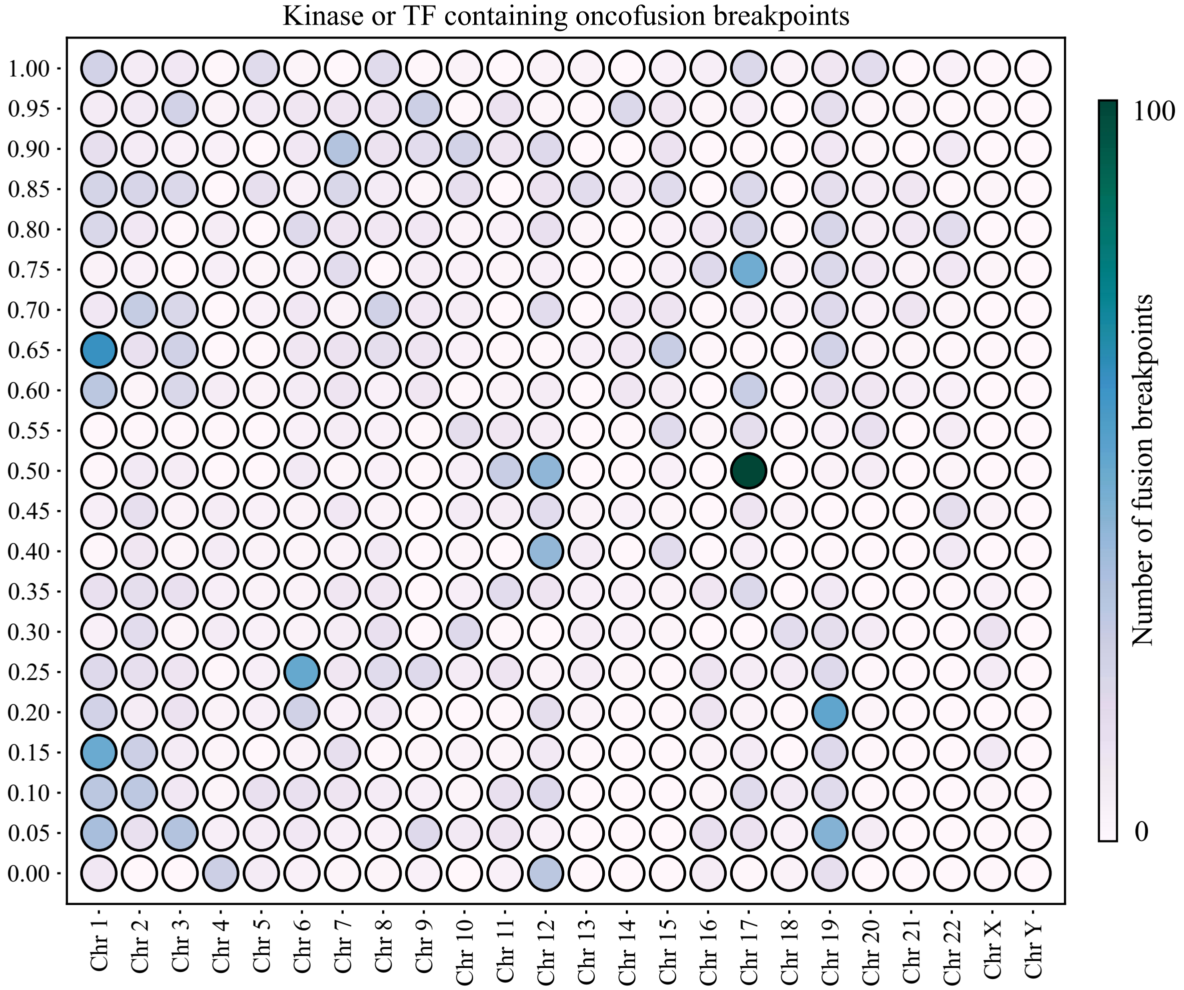

B

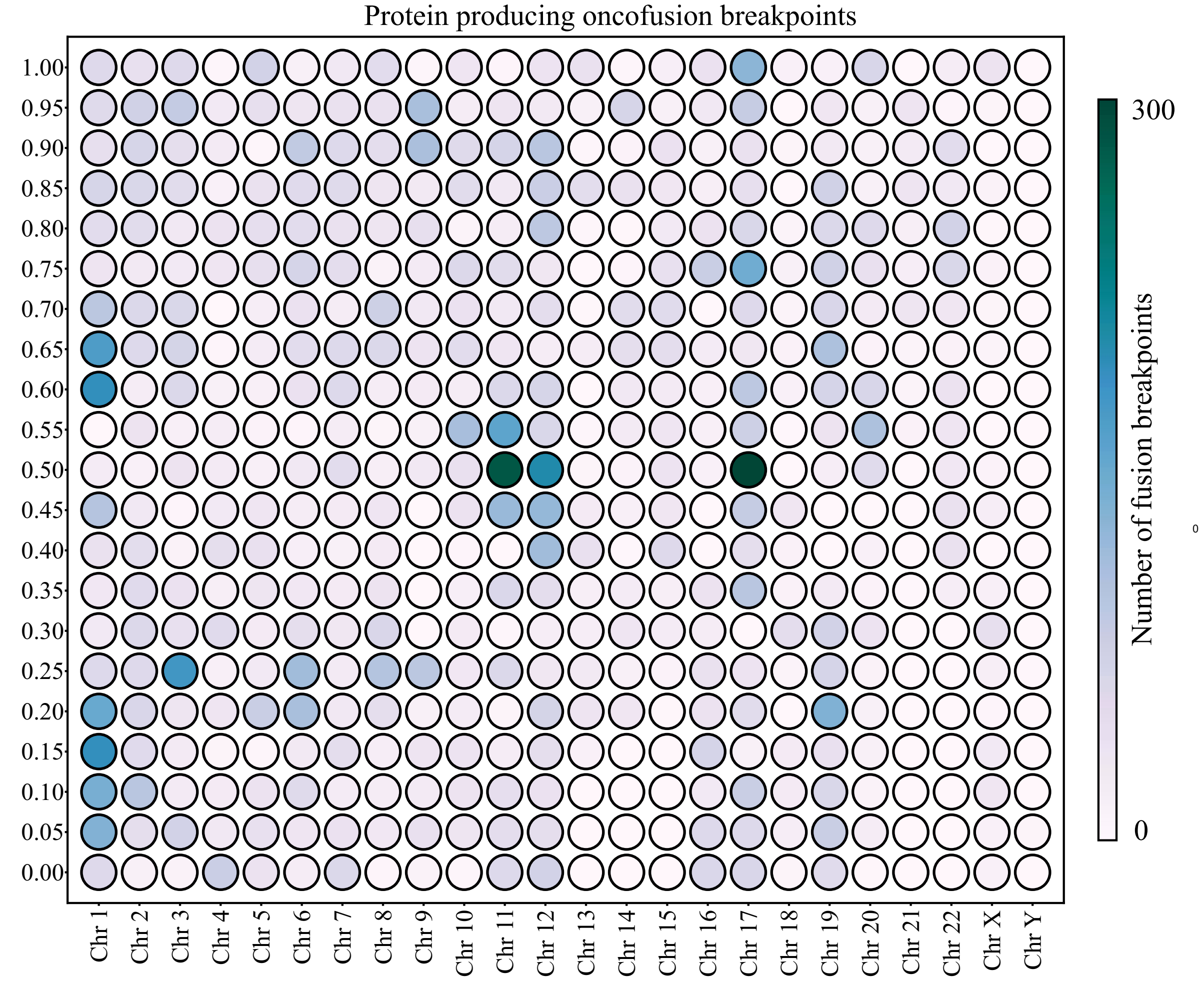

Supplementary figure 3

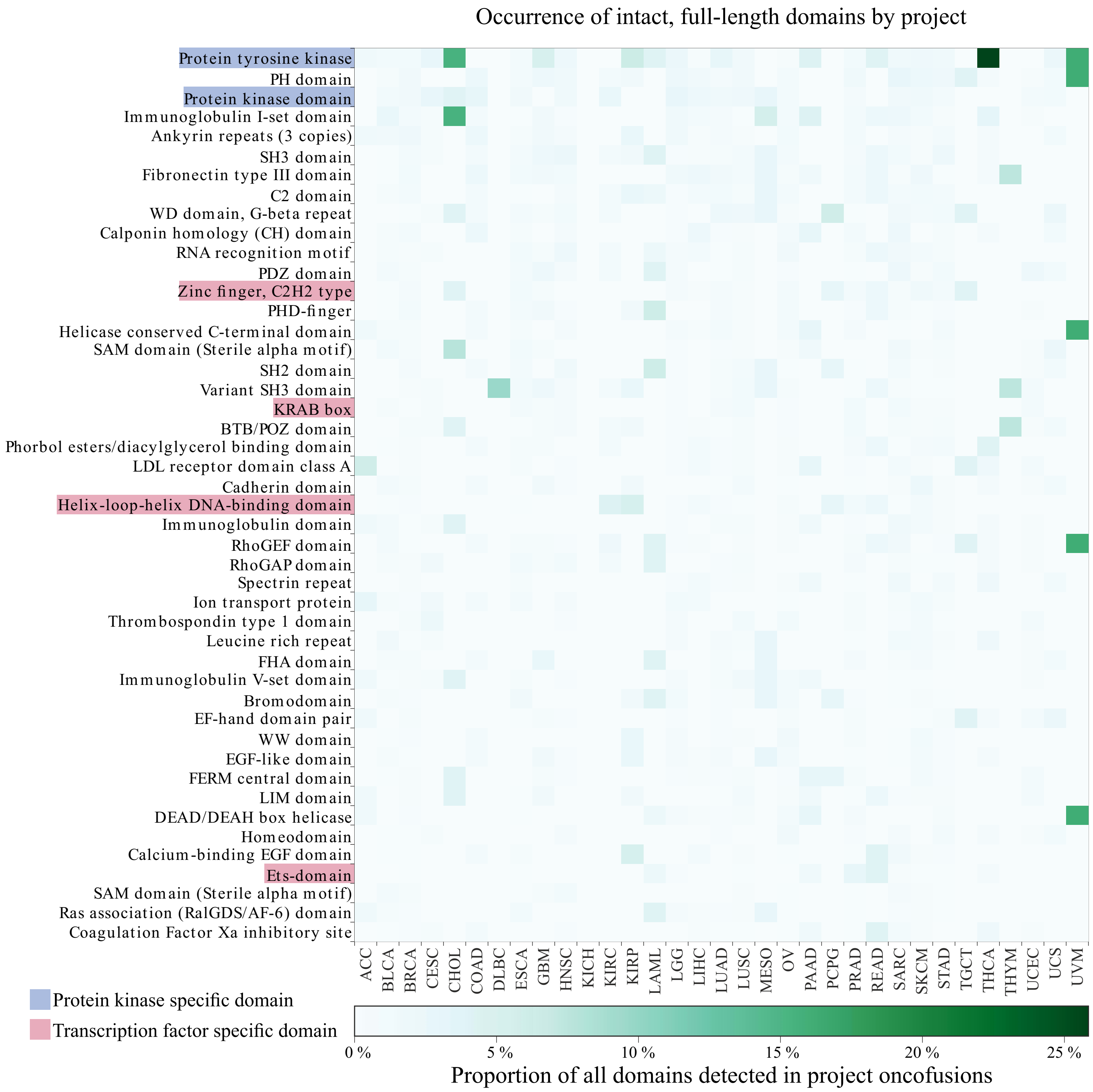

Supplement: Supplementary file 2 — Supplementary information 1. [file 41598_2020_71040_MOESM2_ESM.pdf]
